# A rare case of acute postoperative upper extremity deep vein thrombosis and pulmonary embolus after humeral shaft fixation: A case report and review of the literature

**DOI:** 10.1097/MD.0000000000046655

**Published:** 2025-12-19

**Authors:** Changke Ma, Kehan Tu, Wei Weng, Min Xu, Di Zha, Bin Han

**Affiliations:** a Department of Orthopaedics, Nanjing Luhe People’s Hospital, Yangzhou University, Nanjing, China; b Department of Orthopaedics, The Second Affiliated Hospital of Soochow University, Soochow University, Suzhou, China; c Department of Ultrasonography, Nanjing Luhe People’s Hospital, Yangzhou University, Nanjing, China; d Office of Medical Consortium Administration, Nanjing Luhe People’s Hospital, Yangzhou University, Nanjing, China.

**Keywords:** anticoagulation, case report, complication, pulmonary embolism, upper extremity deep vein thrombosis

## Abstract

**Rationale:**

Upper extremity deep vein thrombosis (DVT) accounts for only 1% to 6% of all DVT cases but is clinically significant due to risks of life-threatening pulmonary embolism and post-thrombotic syndrome. Despite its low incidence, upper extremity deep vein thrombosis warrants heightened clinical vigilance given its frequently occult presentation and potentially devastating sequelae, as underestimation often delays diagnosis and management.

**Patient concerns:**

A 69-year-old woman developed acute-onset chest pain, tightness, and dyspnea on postoperative day 11 following open reduction and internal fixation of a left humeral shaft fracture.The patient reported significant anxiety due to the acute and unexplained nature of her symptoms.

**Diagnoses:**

Doppler ultrasound revealed a hypoechoic thrombus in the left brachial vein. Computed tomographic pulmonary angiography confirmed bilateral pulmonary artery filling defects, indicating pulmonary embolism.

**Interventions:**

The patient received subcutaneous low-molecular-weight heparin (5000 IU twice daily) for 2 weeks as anticoagulation therapy, followed by transition to oral rivaroxaban (20 mg daily). Supplemental oxygen therapy and prophylactic antibiotics were administered concurrently.

**Outcomes:**

Cardiopulmonary symptoms (chest pain, tightness, dyspnea) resolved, left upper limb swelling significantly improved, and serum D-dimer levels markedly declined at discharge. The patient underwent follow-up for 6 months and showed no signs of recurrence.

**Lessons:**

Maintaining a high clinical suspicion is crucial for diagnosing this uncommon syndrome in patients with postoperative humeral fractures who present with persistent or progressive symptoms – including swelling, pain, and cutaneous coolness – particularly in the presence of risk factors such as long-term steroid therapy, indwelling venous catheters and history of cancer. This study aims to enhance awareness of atypical presentations.

## 1. Introduction

Humeral shaft fracture are among the most common upper extremity fractures, accounting for approximately 1% to 3% of all adult fractures and up to 20% of humeral fractures.^[1,2]^ Characteristic clinical manifestations include severe pain, significant swelling, and visible deformity. Only a small proportion of patients develop serious acute traumatic complications such as compartment syndrome or neurovascular injuries. According to a multi-center retrospective analysis spanning 6 hospitals, approximately 7% of such fractures were complicated by neurovascular injury.^[3]^ Most humeral shaft fractures require surgical intervention despite risks of iatrogenic neurovascular injury. The prevalence of nonunion following nonoperative therapy remains 10% to 30% across populations.^[4]^

Fractures predispose patients to deep vein thrombosis (DVT), which occurs predominantly in the lower extremities. Unlike lower limb injuries, upper extremity fractures rarely impair ambulation, contributing to their lower DVT incidence. According to the report, the incidence of upper extremity DVT(UEDVT) ranges from 0.4 to 1 case per 100,000 patients.^[5]^ It may involve any vein from the thoracic inlet to the upper limb, including jugular, subclavian, axillary, brachial, radial, and ulnar veins.^[6]^ DVT and pulmonary embolism (PE), collectively termed venous thromboembolism (VTE), are pathogenetically linked manifestations of the same disease in distinct vascular beds at different temporal stages.

UEDVT often presents with nonspecific symptoms, contributing to diagnostic delays. If untreated, it may cause limb-threatening complications (e.g., ischemia, functional impairment) or PE. A nationwide study in China reported a crude PE incidence of 14.19 per 100,000 population and a mortality rate of 1.00 per 100,000. However, when employing age-standardized rates using the European standard population for more accurate international comparisons, the incidence increased to 19.30 per 100,000, while the mortality rate more than doubled to 2.22 per 100,000.^[7]^ In the United States, PE accounts for 5%–10% of in-hospital deaths.^[8]^ We herein report a rare case of PE secondary to UEDVT following open reduction and internal fixation (ORIF) of a humeral shaft fracture. This case underscores the diagnostic challenges and emphasizes that early intervention in fracture-associated UEDVT is imperative.

## 2. Case report

**Figure 1.**
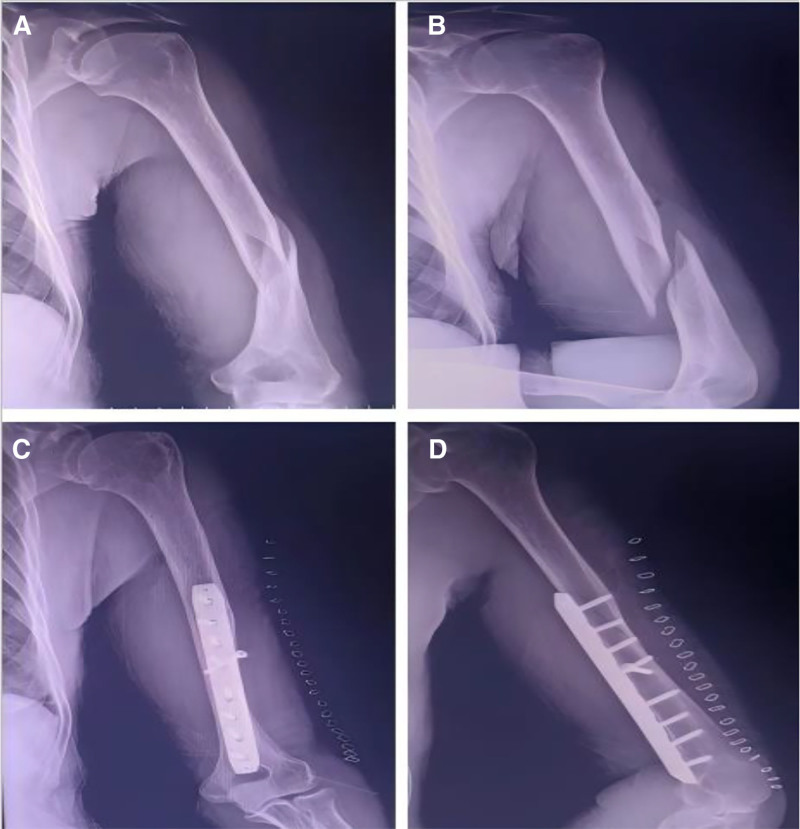
Preoperative and postoperative radiographs of the left humeral shaft fracture. (A and B) Anteroposterior and lateral radiographs of the left humerus display a displaced oblique fracture of the left humeral shaft; (C and D) postoperative radiographs of the left humerus following ORIF demonstrates satisfactory reduction of the fracture with good alignment and position. ORIF = open reduction and internal fixation.

This case report was approved by the Ethics Committee of Nanjing Luhe People’s Hospital and obtained the patient’s informed consent. A 69-year-old woman was admitted to the emergency department with acute-onset chest pain,tightness,and dyspnea that had persisted for 1 hour. Eleven days prior, she had sustained a displaced left humeral shaft fracture in a motor vehicle crash. She subsequently underwent ORIF under brachial plexus block anesthesia, without intraoperative complications. The preoperative D-dimer level was elevated at 1.05 mg/L(reference: 0–0.55 mg/L), and prophylactic anticoagulation with enoxaparin was initiated. However, on postoperative day 2, the patient declined further injections due to pain at the injection site, leading to the discontinuation of anticoagulation based on shared decision-making and her regained ambulatory function. In the absence of new clinical signs suggestive of thrombosis, D-dimer levels were not rechecked. During her recovery, she received prophylactic antibiotics and edema management, and was encouraged to begin early ambulation as well as wrist and elbow functional exercises. Radiographs confirmed satisfactory fracture reduction and implant positioning (Fig. 1). The patient was discharged on postoperative day 5 after an uncomplicated hospital course.Following discharge, she reported progressive left upper limb swelling and mild but worsening pain, for which she did not seek medical attention. While ambulating on postoperative day 11, she developed sudden chest tightness, pain, and dyspnea, prompting transport to the emergency department. Her medical history included hypertension (medication-controlled for 10 years) and rheumatoid arthritis managed with methotrexate and low-dose prednisolone. She underwent unspecified spinal surgery 8 years prior. She reported no family history of tobacco use exposure, venous thromboembolism, or confirmed hereditary thrombophilia.

The patient was a 69-year-old woman with a height of 1.66 m and a weight of 62 kg. Physical examination revealed marked left upper extremity swelling and tenderness in the upper arm. The incision site was clean, without erythema, warmth, or exudate. The radial pulse was diminished. Vital signs included blood pressure 121/93 mm Hg, heart rate 114 bpm, arterial oxygen saturation 90% (reference: 92%–99%), and partial pressure of arterial oxygen 8.9 kPa (reference: 10–13.3 kPa).

Laboratory findings demonstrated an elevated D-dimer level of 7.81 mg/L (reference: 0–0.55 mg/L). This marked elevation strongly suggests an acute and substantial thrombotic process with ongoing fibrinolysis. Other laboratory findings included a normal B-type natriuretic peptide level of 6.47 pg/mL (reference: 0–100 pg/mL), a creatinine level of 67.4 µmol/L (reference: 41–80 µmol/L), and unremarkable cardiac biomarkers: myoglobin 20.5 ng/mL (reference: 14.3–65.8 ng/mL), troponin I 0.003 ng/mL (reference: 0–0.045 ng/mL), and creatine kinase isoenzyme 0.62 ng/mL (reference: 0–5 ng/mL).

**Figure 2.**
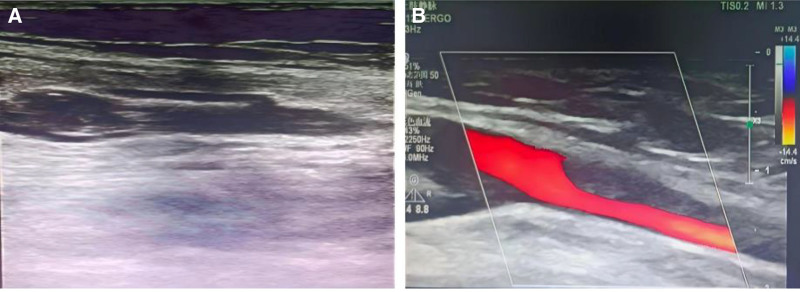
Doppler ultrasound examination at the onset of acute left brachial vein thrombosis. (A) Ultrasound examination of the left brachial vein demonstrating echogenic, expansile thrombus. (B) Doppler examination of the left axillary vein demonstrating filling defects. These findings are consistent with acute upper extremity deep vein thrombosis.

**Figure 3.**
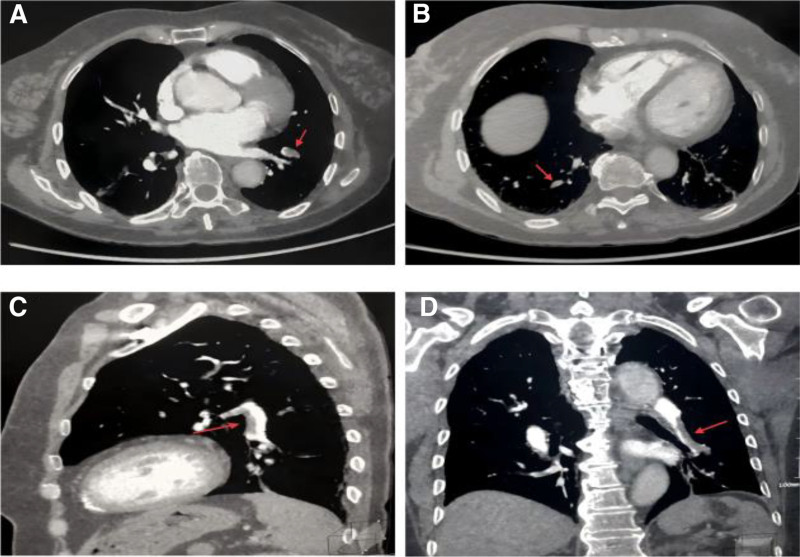
Computed tomographic pulmonary angiography at the onset of pulmonary embolism. (A) Horizontal imaging showed filling defect in the left pulmonary artery branches; (B) horizontal imaging showed filling defect in the right pulmonary artery branches; (C) sagittal imaging showed filling defect in the left pulmonary artery branches; (D) coronal imaging showed filling defect in the left pulmonary artery branches. These findings are consistent with acute subsegmental pulmonary embolism.

Doppler ultrasound (DU) revealed a hypoechoic thrombus in the left brachial vein, extending proximally into the axillary vein, with no involvement of other upper or lower extremity veins (Fig. 2). Computed tomographic pulmonary angiography (CTPA) revealed filling defects in bilateral pulmonary artery branches (Fig. 3), with no associated features of chronic thrombosis, such as mural thickening or calcification. Additionally, the right ventricle was dilated (right/left ventricular diameter ratio of 1.02) without tricuspid annular systolic excursion or significant dysfunction.

The initial differential diagnoses for her acute cardiopulmonary symptoms – including acute coronary syndrome, pneumothorax, and heart failure – were ruled out by clinical evaluation, electrocardiography, and laboratory testing. The diagnosis was ultimately confirmed by CTPA, which showed filling defects indicative of acute subsegmental PE, and confirmed by DU, which identified a proximal UEDVT. These imaging findings were consistent with the elevated D-dimer level, confirming the diagnoses of acute subsegmental PE and proximal UEDVT following left humeral ORIF.

**Figure 4.**
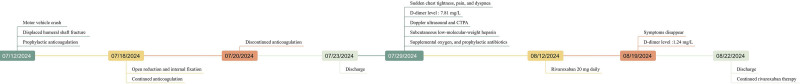
Timeline illustrating acute postoperative UEDVT and PE following humeral shaft fixation. PE = pulmonary embolism, UEDVT = upper extremity deep vein thrombosis

Following admission to the respiratory intensive care unit, the patient underwent formal risk stratification. Given her hemodynamically stable status and a simplified PE severity index score of 1, she was stratified into an intermediate-low risk category. Concurrently, the HAS-BLED score yielded a result of 1, indicating a low risk of major bleeding.These guided a pulmonologist-led multidisciplinary team to initiate a regimen of therapeutic anticoagulation, supplemental oxygen, and prophylactic antibiotics.Subcutaneous low-molecular-weight heparin (5000 IU twice daily) was administered for 14 days.The initial therapeutic dose was selected based on the patient’s body weight of 62 kg, which falls within the standard range for fixed-dose low-molecular-weight heparin in acute VTE, and her normal renal function, thereby permitting full-dose administration without adjustment. After 14 days, the patient was transitioned to rivaroxaban 20 mg daily. By day 21, respiratory symptoms (chest tightness, pain, and dyspnea) and left upper limb swelling had resolved. Serum D-dimer levels decreased significantly to 1.24 mg/L, demonstrating a downward trend. The patient was discharged on day 24 and continued rivaroxaban therapy for 3 months (Fig. 4). The ongoing use of chronic prednisolone was considered a persistent provoking risk factor for VTE recurrence.Given that the need for chronic prednisolone constituted a strong, persistent provoking risk factor for VTE recurrence, and in accordance with guidelines from the American Society of Hematology and the American College of Chest Physicians, the decision was made to extend anticoagulation therapy. During the 6-month follow-up after discharge, the patient remained asymptomatic, with persistently normal serum D-dimer levels.

## 3. Discussion

### 3.1. Risk factors

VTE is categorized as either primary or secondary in origin. Primary VTE, sometimes referred to as spontaneous or idiopathic VTE, arises from inherited hypercoagulable conditions or structural defects that increase susceptibility to thrombus formation. A common example of a structural defect is venous thoracic outlet syndrome, also known as Paget–Schroetter syndrome. This condition is caused by anatomical compression of the subclavian vein at the narrowed thoracic outlet. The resulting repeated mechanical stress triggers a cycle of endothelial injury and repair, eventually leading to UEDVT and fibrotic changes.^[9]^ The other major category of primary VTE causes is genetic hypercoagulability. Among the most frequently encountered inherited thrombophilias are the Factor V Leiden mutation and the prothrombin G20210A gene variant.^[10]^ It is important to note that antiphospholipid syndrome is an acquired, not inherited, thrombophilia and is also a strong risk factor for primary VTE.

Common risk factors for secondary VTE include: advanced age,indwelling venous catheters, surgery, trauma,acquired hypercoagulable states related to malignancy, metabolic syndrome,oral contraceptive use or pregnancy.^[11–13]^ Advanced age independently increases VTE risk, with patients > 75 years exhibiting tenfold higher incidence than younger cohorts.^[11,14]^ Additionally, metabolic syndrome components – hyperglycemia, hypertension, obesity, and dyslipidemia – are prevalent in these patients,^[12,15]^ which collectively promote thrombogenesis by accelerating atherosclerosis, reducing vascular wall elasticity, and increasing blood viscosity. A study by Zhen et al^[7]^ revealed that hypertension, ischemic heart diseases, and heart failure were the 3 most common multimorbidities among patients with PE. Meanwhile, research indicates that UEDVT following fractures of the proximal humerus, humeral shaft, or clavicle is associated with fracture location, conferring an elevated risk.^[16,17]^ Thrombosis predominantly involves the brachial, axillary, and subclavian veins, though pathogenic mechanisms remain incompletely understood^[6,18–21]^ (Table 1). Although the pathogenic mechanisms are incompletely understood, they may be attributed to anatomical vulnerability characterized by high vascular density and tortuous vasculature susceptible to mechanical injury. Advanced age, uncontrolled pain, and misguided activity restrictions frequently lead to prolonged immobility. This reduces blood flow velocity, impairs venous return, and induces swelling. When combined with trauma-induced vascular injury and hypercoagulability, these factors substantially increase VTE risk. Identifying such profiles enables targeted prophylaxis in traumatic upper limb fractures.

**Table 1:** Literature review on UEDVT: examples of recently documented case reports.

Author (year)	Injury type	Treatment of injury	Affected vein	Treatment of UEDVT
Miyake et al, 2023^[12]^	Right clavicle fracture and left distal radius fracture	ORIF	Right subclavian vein	Anticoagulation (Heparin)
Salášek et al, 2022^[11]^	Left clavicle fracture	Nonoperation	Left brachial vein	Anticoagulation (Enoxaparin)
AlRabiah et al, 2022^[14]^	Right shoulder dislocation	Nonoperation	Right cephalic vein and subclavian vein	Anticoagulation (Aspirin)
Jones et al, 2010^[10]^	Left clavicle fracture	Nonoperation	Left subclavian, axillary and brachial veins	Anticoagulation (Heparin)
Strony et al, 2019^[13]^	Left Proximal humerus fracture	ORIF	Left axillary vein	Anticoagulation (Aspirin)

ORIF = open reduction and internal fixation, UEDVT = upper extremity deep vein thrombosis.

In this case, we propose that thrombogenesis was directly associated with the humeral shaft fracture and subsequent ORIF through 2 interconnected risk factors: Excessive traction for fracture exposure and ideal reduction during surgery may have injured the brachial vein, even though the vessel was not directly encountered; Fracture-related hemorrhage combined with surgical trauma induced a hypercoagulable state, further compounded by rheumatoid arthritis and chronic prednisolone use.The confluence of venous stasis from potential injury, a systemic hypercoagulable state, and local vascular trauma created an ideal environment for thrombogenesis, prolonging the exposure of activated platelets and coagulation factors to the injured endothelium and amplifying clot formation through integrated mechanical and biochemical pathways.

In contrast to the typical association of VTE with indwelling catheters or malignancy, this case demonstrates that chronic iatrogenic corticosteroid exposure should be recognized as a primary, independent risk factor. The patient developed VTE following prolonged low-dose prednisone therapy in the absence of other common triggers. This finding highlights that sustained corticosteroid use, even at low doses, warrants consideration as a significant risk factor for VTE, particularly in patients with underlying prothrombotic conditions.

### 3.2. Prothrombotic mechanisms of corticosteroids

The prothrombotic mechanisms associated with chronic corticosteroid use are multifaceted and likely synergistic. Corticosteroids stimulate bone marrow hematopoiesis, increasing platelet production and enhancing aggregation, while also promoting coagulation by elevating fibrinogen levels and shortening prothrombin time; these effects collectively foster a hypercoagulable state. Long-term use also induces dyslipidemia, increases blood viscosity, and causes vasoconstriction, thereby accelerating atherosclerosis. Moreover, corticosteroids can damage vascular endothelial cells, which prompts the release of procoagulant substances and reduces anticoagulant mediators (e.g., nitric oxide, prostacyclin), thus shifting the hemostatic balance toward thrombosis.^[22,23]^ Additionally, Chronic corticosteroid administration leads to fluid retention, elevated intravascular pressure, and various metabolic disruptions, all of which contribute to accelerated atherosclerosis and an increased thrombotic risk.

### 3.3. Symptoms and complications

Common UEDVT manifestations are nonspecific and often lead to delayed diagnosis. Persistent limb swelling, pain, sensory diminution, and decreased skin temperature necessitate immediate UEDVT assessment.The most severe complication is thrombus dislodgement causing massive PE, a potentially fatal event. Symptomatic PE occurs in approximately 12% of UEDVT cases, while 36% develop asymptomatic PE.^[24]^ Acute dyspnea, chest pain, syncope, hemoptysis, tachycardia, or tachypnoea strongly suggest PE, though definitive diagnosis requires CTPA. Long-term sequelae include post-thrombotic syndrome, the most prevalent chronic complication of DVT. Post-thrombotic syndrome manifests as chronic limb pain, swelling, and potential ulceration due to persistent venous obstruction and valvular incompetence. Timely intervention is imperative to prevent these complications.

### 3.4. Examinations

For suspected DVT, DU is the primary diagnostic modality,^[25,26]^ evaluating venous flow and thrombus location with high sensitivity (84%) and specificity (93%).^[27]^ Its noninvasiveness, cost-effectiveness, and technical simplicity render it ideal for routine DVT screening in orthopedic patients. When DU finding is negative but clinical suspicion persists, computed tomography angiography may be warranted. For suspected PE, CTPA is preferred, directly visualizing pulmonary vasculature and thrombi with > 95% sensitivity.^[28]^ Although traditional digital subtraction angiography remains the gold standard, its invasiveness carries inherent risks such as infection and vascular injury. Therefore, current guidelines reserve digital subtraction angiography for selected cases with persistent diagnostic uncertainty after noninvasive testing.

Laboratory findings may include nonspecific leukocytosis. D-dimer testing demonstrates high sensitivity but low specificity for VTE, with high negative predictive value. A negative result can largely exclude VTE.^[29,30]^ However, elevated D-dimer levels correlate with multiple conditions including disseminated intravascular coagulation, malignancy, preeclampsia, infection, and trauma, and its specificity is further reduced in advanced age. Thus, while clinically useful for VTE risk stratification, D-dimer interpretation requires correlation with confirmatory imaging such as DU or CTPA. Arterial blood gas analysis serves as a rapid adjunctive screening tool for PE, frequently revealing hypoxemia secondary to pulmonary vascular obstruction, demonstrated by a reduction in PaO₂.

In this case, a critical limitation was the failure to recognize the patient’s chronic steroid use as a significant risk factor. This omission resulted in the absence of perioperative anticoagulation and postoperative D-dimer monitoring. Consequently, an opportunity for early intervention was likely missed, ultimately leading to the development of UEDVT and PE. This underscores the need for a more vigilant monitoring protocol in high-risk patients. For patients with recognized risk factors, perioperative dynamic monitoring of D-dimer and coagulation function is essential for the early detection of a hypercoagulable state and initiation of anticoagulant therapy.

### 3.5. Preventions and treatments

A well-established consensus exists for prophylactic treatment against DVT following major lower extremity orthopedic surgeries, typically including pharmacological anticoagulation with agents such as warfarin, rivaroxaban, and low-molecular-weight heparin, early mobilization, and dietary optimization. Preoperative interventions also include limb elevation and ankle exercises to improve venous return. Postoperatively, intermittent pneumatic compression is routinely applied to enhance venous flow.Dietary guidance emphasizes adequate hydration to prevent hemoconcentration and the avoidance of hyperlipidemic foods, hyperosmolar solutions, and tobacco. Routine DVT screening and prophylactic anticoagulation remain controversial for upper extremity fractures due to a lack of high-quality evidence to guide practice, compounded by the substantially lower incidence of UEDVT compared to lower extremity DVT and the even rarer occurrence of PE.

In patients with upper extremity fractures complicated by UEDVT, priority should be given to prompt thrombus management over surgical intervention. Primary treatment options include anticoagulation or thrombolysis. Thrombolytic therapy is the first-line approach for hemodynamically unstable patients to rapidly restore perfusion, while anticoagulation is generally preferred for hemodynamically stable patients.^[31,32]^ These interventions prevent embolization, reduce propagation, and promote dissolution of existing thrombi while protecting vascular endothelium and decreasing adhesiveness of residual wall-adherent thrombi.Following successful thrombolysis, standard anticoagulation should continue with stringent coagulation monitoring.If thrombus progression persists (e.g., enlargement or free-floating morphology indicating detachment risk), superior vena cava filter placement may be considered; however, this carries significant risks and requires careful patient selection, despite its efficacy for thrombi > 3 mm. For PE cases refractory or contraindicated to thrombolysis, catheter-directed pulmonary thrombectomy may be indicated. Right ventricular dysfunction may necessitate adjunctive diuretics and inotropes to reduce the load.Notably, standardized UEDVT management protocols remain lacking, with considerable controversy regarding optimal post-traumatic anticoagulation timing.

Increasing life expectancy has led to a growing population of patients with multi-organ damage. In such cases, anticoagulation therapy should be personalized based on individual patient factors, including age, weight, comorbidities, and eGFR, to guide drug selection and dosage adjustment. Warfarin is not preferred due to its narrow therapeutic index and complex dosing, which are particularly challenging to manage in patients with renal impairment. Instead, novel oral anticoagulants with lower renal clearance and improved safety profiles are recommended. For example, edoxaban demonstrates a favorable renal safety profile, offering a clear net clinical benefit in this population. It is not metabolized by CYP450 enzymes and does not adversely affect renal function, making it a preferred therapeutic option for older adults and those with renal impairment.^[33–35]^ In contrast, dabigatran is predominantly renally excreted and more impacted by declining renal function.^[36–38]^ Thus, edoxaban is preferable to both warfarin and dabigatran in patients with moderate to severe renal impairment. For patients at high bleeding risk, potential drug interactions require careful attention. Warfarin interacts with numerous medications, while novel oral anticoagulants exhibit fewer interactions. Therefore, in elderly patients with renal impairment or elevated bleeding risk, agents with a strong safety profile and minimal drug interactions should be prioritized, accompanied by regular monitoring of renal function and coagulation indicators.

## 4. Conclusion

In conclusion, this postoperative VTE following ORIF of a humeral fracture was likely triggered by intraoperative vascular injury from excessive traction, compounded by chronic prednisolone use. Diagnosis was confirmed by characteristic clinical symptoms and imaging evidence of thrombosis, along with elevated D-dimer levels. Follow-up after anticoagulation showed significant symptom improvement and normalized D-dimer levels, underscoring the value of prompt treatment. We recommend maintaining a high clinical suspicion for VTE in postoperative humeral fracture patients with persistent limb symptoms, particularly those on chronic steroid therapy, and performing early DU and D-dimer examinations to facilitate timely diagnosis and intervention.

## Author contributions

**Conceptualization:** Changke Ma.

**Data curation:** Kehan Tu, Wei Weng.

**Investigation:** Bin Han.

**Methodology:** Kehan Tu.

**Project administration:** Changke Ma.

**Resources:** Changke Ma.

**Supervision:** Changke Ma, Di Zha.

**Validation:** Bin Han.

**Writing – original draft:** Kehan Tu, Min Xu.

**Writing – review & editing:** Changke Ma.

## References

[1] Guo J Ma H. Different treatment for humeral shaft fractures: a network meta-analysis. Medicine (Baltim). 2025;104:e40948. 10.1097/MD.0000000000040948 PMC11749579 39833039

[2] Pidhorz L. Acute and chronic humeral shaft fractures in adults. Orthop Traumatol Surg Res. 2015;101:S41–9. 25604002 10.1016/j.otsr.2014.07.034

[3] Claessen FM Peters RM Verbeek DO Helfet DL Ring D. Factors associated with radial nerve palsy after operative treatment of diaphyseal humeral shaft fractures. J Shoulder Elbow Surg. 2015;24:e307–11. 26341025 10.1016/j.jse.2015.07.012

[4] Suter C Mattila H Ibounig T. Prediction of humeral shaft fracture healing using the Radiographic Union Score for HUmeral Fractures (RUSHU). Bone Jt Open. 2024;5:962–70. 39489162 10.1302/2633-1462.511.BJO-2024-0134.R1 PMC11531895

[5] Kucher N. Clinical practice. Deep-vein thrombosis of the upper extremities. N Engl J Med. 2011;364:861–9. 21366477 10.1056/NEJMcp1008740

[6] AlRabiah AA Kadi AT Al Musallam LI Aldawood AA Alshowihi SS. Acute upper extremity vein thrombosis in recurrent shoulder dislocation. Cureus. 2022;14:e31488. 36532933 10.7759/cureus.31488 PMC9749872

[7] Zhen K Tao Y Xia L; National VTE Prevention Program. Epidemiology of pulmonary embolism in China, 2021: a nationwide hospital-based study. Lancet Reg Health West Pac. 2024;54:101258. 39759425 10.1016/j.lanwpc.2024.101258 PMC11699474

[8] Schleyer AM Schreuder AB Jarman KM Logerfo JP Goss JR. Adherence to guideline-directed venous thromboembolism prophylaxis among medical and surgical inpatients at 33 academic medical centers in the United States. Am J Med Qual. 2011;26:174–80. 21490270 10.1177/1062860610382289

[9] Modi BP Chewning R Kumar R. Venous thoracic outlet syndrome and Paget-Schroetter syndrome. Semin Pediatr Surg. 2021;30:151125. 34930589 10.1016/j.sempedsurg.2021.151125

[10] Hendler MF Meschengieser SS Blanco AN. Primary upperextremity deep vein thrombosis: high prevalence of thrombophilic defects. Am J Hematol. 2004;76:330–7. 15282664 10.1002/ajh.20131

[11] Oger E. Incidence of venous thromboembolism: a community-based study in Western France. EPI-GETBP Study Group. Groupe d’Etude de la Thrombose de Bretagne Occidentale. Thromb Haemost. 2000;83:657–60. 10823257

[12] Dua A Desai SS Nodel A Heller JA. The impact of body mass index on lower extremity duplex ultrasonography for deep vein thrombosis diagnosis. Ann Vasc Surg. 2015;29:1136–40. 26004960 10.1016/j.avsg.2015.03.038

[13] Koethe Y Bochnakova T Kaufman CS. Upper extremity deep venous thrombosis: etiologies, diagnosis, and updates in therapeutic strategies. Semin Intervent Radiol. 2022;39:475–82. 36561939 10.1055/s-0042-1757937 PMC9767760

[14] Bassa B Little E Ryan D. VTE rates and risk factors in major trauma patients. Injury. 2024;55:111964. 39481253 10.1016/j.injury.2024.111964

[15] Song K Rong Z Yao Y Shen Y Zheng M Jiang Q. Metabolic syndrome and deep vein thrombosis after total knee and hip arthroplasty. J Arthroplasty. 2016;31:1322–5. 26989028 10.1016/j.arth.2015.12.021

[16] Prensky C Urruela A Guss MS Karia R Lenzo TJ Egol KA. Symptomatic venous thrombo-embolism in low-energy isolated fractures in hospitalised patients. Injury. 2013;44:1135–9. 23684349 10.1016/j.injury.2013.04.018

[17] Nayar SK Kuwabara AM Flores JM Osgood GM LaPorte DM Shafiq B. Venous thromboembolism in upper extremity fractures. J Hand Surg Asian Pac Vol. 2018;23:320–9. 30282549 10.1142/S2424835518500303

[18] Jones RE McCann PA Clark DA Sarangi P. Upper limb deep vein thrombosis: a potentially fatal complication of a clavicle fracture. Ann R Coll Surg Engl. 2010;92:W36–8. 20529480 10.1308/147870810X12699662980358 PMC5696955

[19] Salášek M Pavelka T Weisová D. Deep venous thrombosis after conservative treatment of clavicular fracture in COVID-19 negative children: two case reports. Acta Chir Orthop Traumatol Cech. 2022;89:435–40. 36594691

[20] Miyake Y Abe T Suekane A Goan A Ameda T Ochiai H. Venous thoracic outlet syndrome with an upper extremity deep vein thrombosis caused by a dislocated clavicle fracture: a case report. Am J Case Rep. 2023;24:e939250. 37431093 10.12659/AJCR.939250 PMC10348390

[21] Strony J Chang G Krieg JC. Upper-extremity deep venous thrombosis following a fracture of the proximal humerus: an orthopaedic case report. Case Rep Orthop. 2019;2019:6863978. 31781453 10.1155/2019/6863978 PMC6875200

[22] Simion C Campello E Bensi E. Use of glucocorticoids and risk of venous thromboembolism: a narrative review. Semin Thromb Hemost. 2021;47:654–61. 33893633 10.1055/s-0040-1722270

[23] Rastoder E Sivapalan P Eklöf J. Systemic corticosteroids and the risk of venous thromboembolism in patients with severe COPD: a nationwide study of 30,473 outpatients. Biomedicines. 2021;9:874. 34440079 10.3390/biomedicines9080874 PMC8389624

[24] Margey R Schainfeld RM. Upper extremity deep vein thrombosis: the oft-forgotten cousin of venous thromboembolic disease. Curr Treat Options Cardiovasc Med. 2011;13:146–58. 21271312 10.1007/s11936-011-0113-1

[25] Parv F Tudoran C Parv C. Aetiological, clinical and therapeutic prognostic factors for the evolution of deep vein thrombosis followed up with serial venous Doppler ultrasound. Intern Med J. 2023;53:409–15. 35050533 10.1111/imj.15693

[26] Needleman L Feld R. Peripheral venous ultrasound. Radiol Clin North Am. 2025;63:165–78. 39510660 10.1016/j.rcl.2024.08.001

[27] Di Nisio M Van Sluis GL Bossuyt PM Büller HR Porreca E Rutjes AWS. Accuracy of diagnostic tests for clinically suspected upper extremity deep vein thrombosis: a systematic review. J Thromb Haemost. 2010;8:684–92. 20141579 10.1111/j.1538-7836.2010.03771.x

[28] Zantonelli G Cozzi D Bindi A. Acute pulmonary embolism: prognostic role of computed tomography pulmonary angiography (CTPA). Tomography. 2022;8:529–39. 35202207 10.3390/tomography8010042 PMC8880178

[29] Philbrick JT Heim S. The d-dimer test for deep venous thrombosis: gold standards and bias in negative predictive value. Clin Chem. 2003;49:570–4. 12651808 10.1373/49.4.570

[30] Lv B Xue F Shen YC Hu F-B Pan M-M. Pulmonary thromboembolism after distal ulna and radius fractures surgery: a case report and a literature review. World J Clin Cases. 2021;9:197–203. 33511185 10.12998/wjcc.v9.i1.197 PMC7809660

[31] Konstantinides SV. 2014 ESC guidelines on the diagnosis and management of acute pulmonary embolism. Eur Heart J. 2014;35:3145–6. 25452462 10.1093/eurheartj/ehu393

[32] Kearon C Akl EA Ornelas J. Antithrombotic therapy for VTE disease: CHEST guideline and expert panel report. Chest. 2016;149:315–52. 26867832 10.1016/j.chest.2015.11.026

[33] Wang Y Li L Wei Z. Efficacy and safety of renal function on edoxaban versus warfarin for atrial fibrillation: a systematic review and meta-analysis. Medicines (Basel). 2023;10:13. 36662497 10.3390/medicines10010013 PMC9861612

[34] Pathak R Pandit A Karmacharya P. Meta-analysis on risk of bleeding with apixaban in patients with renal impairment. Am J Cardiol. 2015;115:323–7. 25527282 10.1016/j.amjcard.2014.10.042

[35] Zhou LY Yin WJ Zhao J. A novel creatinine-based equation to estimate glomerular filtration rate in chinese population with chronic kidney disease: implications for DOACs dosing in atrial fibrillation patients. Front Pharmacol. 2021;12:615953. 33679397 10.3389/fphar.2021.615953 PMC7933563

[36] Lin S Wang Y Zhang L Guan W. Dabigatran must be used carefully: literature review and recommendations for management of adverse events. Drug Des Devel Ther. 2019;13:1527–33. 10.2147/DDDT.S203112 PMC6511609 31190734

[37] Hohnloser SH Steg PG Oldgren J; RE-DUAL PCI Steering Committee and Investigators. Renal function and outcomes with dabigatran dual antithrombotic therapy in atrial fibrillation patients after PCI. JACC Cardiovasc Interv. 2019;12:1553–61. 31439336 10.1016/j.jcin.2019.05.050

[38] Li X Zuo C Ji Q Wang Z Lv Q. Impact of renal function on effectiveness and safety associated with low dose dabigatran in non-valve atrial fibrillation patients after catheter ablation. Front Cardiovasc Med. 2021;8:762872. 34778414 10.3389/fcvm.2021.762872 PMC8581241

